# WHO key access antibiotics price, availability and affordability in private sector pharmacies in Pakistan

**DOI:** 10.1186/s12962-021-00263-x

**Published:** 2021-02-16

**Authors:** Zikria Saleem, Hamid Saeed, Zunaira Akbar, Amna Saeed, Saleha Khalid, Laiba Farrukh, Aleena Irfan, Azka Anam, Mohamed Azmi Hassali, Huma Rasheed, Zaheer-Ud-Din Babar

**Affiliations:** 1 Department of Pharmacy Practice, Faculty of Pharmacy, The University of Lahore, Lahore, Pakistan; 2 University College of Pharmacy, University of the Punjab, Allama Iqbal Campus, Lahore, Pakistan; 3 Riphah Institute of Pharmaceutical Sciences, Riphah International University, Lahore, Pakistan; 4 Department of Pharmacy Administration and Clinical Pharmacy, School of Pharmacy, Jiaotong University, Xi’an, Shaanxi China; 5 Center for Drug Safety and Policy Research, Xian Jiaotong University, Xi’an, Shaanxi China; 6 The Global Health Institute, Xi’an Jiaotong University, Xi’an, Shaanxi China; 7 Shaanxi Centre for Health Reform and Development Research, Xi’an, China; 8 School of Pharmaceutical Sciences, Universiti Sains Malaysia, George Town, Malaysia; 9 Institute of Pharmaceutical Sciences, University of Veterinary and Animal Sciences, Lahore, Pakistan; 10 Department of Pharmacy, University of Huddersfield, Queensgate, Huddersfield, HD1 3DH UK

**Keywords:** Key access antibiotics, Price, Availability, Affordability

## Abstract

**Background:**

Poor availability and unaffordability of key access antibiotics may increase antimicrobial resistance in the community by promoting inappropriate antibiotic selection and abridged therapy compliance.

**Objective:**

To check the prices, availability, and affordability of the World Health Organization (WHO) key access antibiotics in private sector pharmacies of Lahore, Pakistan.

**Methodology:**

A survey of WHO key access antibiotics from WHO essential medicine list 2017 was conducted in private sector pharmacies of 4 different regions of Lahore employing adapted WHO/HAI methodology. The comparison of prices and availability between originator brands (OB) and lowest price generics (LPG) were conducted followed by the effect of medicine price differences on patient’s affordability. The data were analyzed using a preprogrammed WHO Microsoft excel workbook.

**Results:**

The mean availability of OB products was 45.20% and the availability of LPGs was 40.40%. The OBs of co-amoxiclav, clarithromycin and metronidazole and LPGs of azithromycin and ciprofloxacin were easily available (100%) in all private sector pharmacies. Whereas, antibiotics like chloramphenicol, cloxacillin, nitrofurantoin, spectinomycin, and cefazolin were totally unavailable in all the surveyed pharmacies. The OBs and LPGs with high MPRs were ceftriaxone (OB; 15.31, LPG; 6.38) and ciprofloxacin (OB; 12.42, LPG; 5.77). The median of brand premium obtained was 38.7%, which varied between the lowest brand premium of 3.97% for metronidazole and highest for ceftriaxone i.e. 140%. The cost of standard treatment was 0.5 day’s wage (median) if using OB and 0.4 day’s wage (median) for LPG, for a lowest paid unskilled government worker. Treatment with OB and LPG was unaffordable for ciprofloxacin (OB; 2.4, LPG; 1.1) & cefotaxime (OB; 12.7, LPG; 8.1).

**Conclusion:**

There is dire need to properly implement price control policies to better regulate fragile antibiotic supply system so that the availability of both OB and LPG of key access antibiotics should be increased. The prices could be reduced by improving purchasing efficiency, excluding taxes and regulating mark-ups. This could increase the affordability of patients to complete their antibiotic therapy with subsequent reduction in antimicrobial resistance.

## Background

Medicines constitute the major expenditure of household budget in the developing countries. Medicine prices, availability and affordability are pivotal in promoting access to medicines in the developing countries [1]. It is important to explore and understand the reasons behind the medicine price mechanisms as they define the affordability for patients [2]. Owing to higher medicine prices, it becomes unaffordable for the people living in the low-income countries to buy medicines out of their pockets [3], which compelled them to skip their medical treatment—leading to increase rate of morbidity and mortality [4]. Since, majority of the health care expenses, including the cost of medicines, are covered by the out-of-pocket expenses, the higher medicines prices majorly contribute in pushing people towards poverty. In this epoch of growing infectious diseases, where a sizable portion of the burden is shared by LMICs, we are totally dependent on antibiotics for the treatment of several life-threatening infectious diseases [5]. Howbeit an incomplete treatment with an antibiotic, due to any reason, can lead to the development of antimicrobial resistance (AMR) [6]. AMR is now international concern [7, 8]. Concurrently, with the growing level of AMR to first-line antibiotics, the second or third line choice of antibiotics become more costlier, which are often unaffordable to many in low and middle-income countries (LMICs) [9]. WHO recognized the gap in 1977, and published its first essential medicine list (EML) that revolutionized the public health practices [10]. The EML also highlighted the need and concernment of some medicines over the others and the issues related to their unavailability to the people in developing countries. Since then, the EML has increased in size and has become an evidence-based process, covering efficacy, safety and cost-effectiveness [10].

According to WHO, the availability of EML drugs in LMICs is only 35% in the public sector facilities and 66% in the private sector, where people in low-income countries spend 20–60% on health, while in the developed countries the spending is 18% [11]. In Pakistan, the situation is more or less same probably due to recurrent deficiency and unavailability of essential medicines in government health facilities [11]. Thus, among other reasons, the non-availability of medicines and even doctors in the government health facilities, compel the patient, almost 67%, to consult private physicians or a private pharmacy, almost 3 times more often from a private pharmacy rather than from a basic unit or rural health center [12].

In 2017, WHO has divided the EML into Access, Watch and Reserve (AWaRe) groups as an antimicrobial stewardship strategy to fight against AMR [13]. This was the commitment asked by the WHO from the member countries regarding the availability, affordability and quality of these antibiotics. Twenty-nine different medicines are included in the access group, out of which, 7 antibiotics, including cefixime, ceftriaxone, cefotaxime, piperacillin and tazobactam, and additionally, azithromycin, ciprofloxacin, clarithromycin and vancomycin are also shared with Watch group. The latter medicines are the ones with higher potential of developing antimicrobial resistance and are regarded as critically important medicines for the human use – altogether shared as ACCESS medicines, thus are priority medicines with regards to the availability, affordability and quality [14]. This list acts as a model list and the member countries are expected to make up a similar list, termed as a National Essential Medicines List based on the similar rationale and guidelines, keeping in view the pharmaceutical needs of their own population. Likewise, Pakistan has its NEML updated in 2018 which differ from WHO-EML for only a few molecules [15].

Moreover, in Pakistan, the flaccid implementation of dispensing controls, such as dispensing of prescription only medicines (PoM) strictly on presenting legitimate prescription, may further complicate the status of the poor availability and affordability of essential medicines [16, 17]. A free access to Watch and Reserve medicine can lead to over use or misuse of medicines. The situation further becomes arduous by unregulated marketing, placing high prescribing pressures on relatively high cost medicines and restricting use of other medicines. It is also important to understand that the WHO member states commitment to the Universal Health coverage more likely to promote the availability and access to the essential medicines. Certain national legislative support is also present with respect to the shortages of medicines. According to The Drug Act 1976, no pharmaceutical manufacturer should involve in shortages of drugs without prior approval of the Drug Regulatory Authority of Pakistan (DRAP) [18].

The objective of our study was to investigate the prices, availability and affordability of key access antibiotics from WHO essential medicine list at private pharmacies located in different regions of Lahore, Pakistan.

## Methodology

### Study design and center

A cross-sectional survey was conducted by enrolling 16 private sector pharmacies from 4 different regions of Lahore, Pakistan. The study followed a customized WHO/HAI methodology by consulting manual on “Measuring medicine prices, availability, affordability and price components” [19]. The WHO/HAI methodology requires the survey region to be divided into six areas based on their levels of administration defined by the government (e.g. provinces or cities). In our study, Lahore division was taken as a survey region which has four districts in it (Lahore, Kasur, Sheikhupura and Nankana Sahib). So, we included all the four areas for survey. According to the standard methodology, in each survey area (i.e. district), one biggest public sector hospital and its one nearby pharmacy (within 10 km distance from the hospital) are selected as survey anchor. Four more public sector hospitals and their nearby private pharmacies are selected randomly, that are situated within 3 h drive from the survey anchor. We compiled a complete list of public sector hospitals in each district, not all districts under survey had 5 public hospitals, so we selected four hospitals in each district and hence four pharmacies in each district, making up a total of 16 pharmacies. This sampling technique has also been used formerly [16]. Although WHO/HAI methodology includes both public and private sector facilities to be included in the surveys, yet in Pakistan the medicines are provided free of cost in the public sector facilities, thus in our study, we focused only on private sector data i.e. retail pharmacies, where patients are paying out of pocket to get the medicines. Only registered privately owned retail/community pharmacies were included in the survey.

### Selection of antibiotics

Only key access group of antibiotic from WHO EML 2017 were selected for inclusion in the survey [20]. Each medicine’s dosage form, strength, pack size for both originator brand (OB) and lowest price generic (LPG) were defined.

### Data collection and analysis

Data on the availability and prices (maximum retail prices) of the defined dosage form, strength and pack size of each medicine was collected for both the OB and the LPG using a standardized data collection form. MRPs are fixed by Drug Regulatory Authority of Pakistan (DRAP) [21].

OBs are defined as the product that was first authorized worldwide for marketing, normally as a patented product, on the basis of the documentation of its efficacy, safety and quality, according to requirements at the time of authorization. LPGs are the lowest priced alternates available/registered in the country or the region of survey. For the purpose of this study these generics were accessed using the survey carried out for product availability. LPGs recorded in the study should not be confused with the lowest possible priced generics that can be made available as per the market authorization status in the country/region of study.

Availability of each medicine was marked after checking the stock physically. The selected pharmacies were surveyed from 16 February 2018 to 16 March 2018. The data was fed into a preprogrammed WHO Microsoft excel workbook [22], which already had different sheets and built-in formulas out of which we used only 4 sheets; home page, international medicine reference price data sheet, field data consolidation including private sector patient prices sheet and standard treatment affordability sheet. On the first day of data collection 16 February 2018, 1 US dollar was equal to 110.300 PKR and all the prices were converted into US dollars using OANDA currency converter, as the workbook uses US dollars as the currency for recording references. The data was entered into the international medicine reference price data sheet and rechecked using double entry feature, ensuring correct data entry. Percentage availability, summary tables and median price ratio were calculated automatically by the workbook itself. The median price ratio (MPR) is the ratio of the median local unit price (in the local currency) to a unit of international price. The IRPs are the medians of recent procurement or tender prices offered by predominantly not-for-profit suppliers to developing countries for multi-source products. These are published by the Management Sciences for Health and are obtained from the 2015 International Medical Products Price Guide [23].

According to the WHO/HAI methodology, a median price ratio (MPR). MPR indicates how much the local medicine price is higher or lower than the international prices. Normally, an MPR of 1 or less is taken as efficient procurement in the public sector, while below 3 is considered efficient for the private sector [2, 24].

Band premium (BP) value was calculated by measuring the difference between brand median price and generic median price. The medicine affordability was measured by taking into account, the total cost of medication for the prescribed duration of a treatment for a particular disease and the average wage of the lowest paid unskilled government worker of a particular country. Affordability was calculated using standard treatment affordability sheet. Affordability of 6 diseases and infections common in Pakistan, was checked which covered a total of 5 key access antibiotics. The wage of a lowest paid government worker was considered for calculating the affordability of medicine, as used by all previous studies conducted by following the WHO/HAI methodology [16]. The affordability was checked for two threshold levels of wages (Table 5) i.e. minimum and maximum daily wages for a lowest paid government worker, which were PKR 304.33 and PKR 594.33 respectively [25]. The indications, duration of treatment and medicines for respective indications were entered [6] and the excel sheet automatically calculated the median treatment price and a number of daily wages for the complete treatment. More number of wages spent for any treatment depicted unaffordability of that treatment for the patients.

## Results

In the private sector, the mean availability of OB product was 45.20% and the availability of LPG was 40.40%. Availability of antibiotics was determined using percentile range for the surveyed pharmacies. In OB category, only 3 out of 26 surveyed medicines, were in 100 percentile, 5 were in 25 percentile, 3 were in 25–50 percentile, 1 antibiotic was in 51–75 percentile, 7 antibiotics were in 75–99 percentile, while 7 antibiotics Cefazolin, chloramphenicol, cloxacillin, gentamicin, nitrofurantoin, phenoxymethylpenicillin, spectinomycin were not available in any of the pharmacies enrolled in the study. For LPG category, only 2 antibiotics were in 100 percentiles, 2 antibiotics were in less than 25 percentiles, 6 antibiotics were in 25 to 50 percentile, 6 antibiotics were in 51 to 75% percentile, 3 antibiotics were in 75 to 99 percentile, while the above mentioned 7 antibiotics were totally unavailable in any of the pharmacies surveyed in the study (Tables 1 and 2). The OBs of co-amoxiclav, clarithromycin and metronidazole and LPGs of azithromycin and ciprofloxacin were easily available in all private sector pharmacies. Whereas, OBs and LPGs of 6antibiotics including cefazolin, chloramphenicol, cloxacillin, nitrofurantoin, phenoxymethyl penicillin and spectinomycin were not available in any of the surveyed pharmacies (Tables 1 and 2). Moreover, gentamicin among OBs and clindamycin among LPGs were also not available.

**Table 1:** Availability of individual antibiotics in surveyed pharmacies

Availability (Percentiles)	Originator Brands	Lowest Price Generics
0%	Cefazolin, chloramphenicol, *Cloxacillin,* gentamicin, nitrofurantoin, phenoxymethylpenicillin, spectinomycin	Cefazolin, chloramphenicol, nitrofurantoin, *Cloxacillin, *phenoxymethylpenicillin,clindamycin, spectinomycin
< 25%	Amikacin, *Azithromycin*, *Cefixime*, *Meropenem,,* Benzyl penicillin	Ampicillin, sulfamethoxazole + trimethoprim
25–50%	Doxycycline, *Piperacillin + Tazobactum*, vancomycin	Amoxicillin, Cefalexin, *Cefotaxime,* Gentamicin, Metronidazole, Benzyl penicillin
51–75%	Sulfamethoxazole + trimethoprim	Amikacin, doxycycline, *Meropenem*, *Piperacillin + Tazobactum*, Vancomycin, co-amoxiclav
75%–99%	Ampicillin, *Cefotaxime, Ceftriaxone, Ciprofloxacin*, Clindamycin, Cefalexin, Amoxicillin	*Cefixime, Ceftriaxone, Clarithromycin*
100%	Co-amoxiclav, *Clarithromycin,* Metronidazole	*Azithromycin, Ciprofloxacin,*

Antibiotics in italics are entered in WHO EML as both WACTCH and ACCESS group medicines

**Table 2:** Mean percentage availability of antibiotics

Antibiotics	Originator Brands (%)	Lowest Price Generics (%)
All antibiotics	45.20	40.40
Aminoglycosides	4.2	33.6
Carbapenems	18.8	*56.3*
Cephalosporins	*53.76*	47.5
Quinolones	*93.8*	*100*
Penicillins	46.44	25.91
Macrolides	*68.76*	*64.6*
Tetracyclines	37.5	*62.5*
Nitromidazoles	*100*	25
Sulfonamides	*75*	12.5
Glycopeptide proteins	37.5	*56.3*

The individual median price ratios of all drugs are listed in Table 3. The OBs and LPGs with high MPRs include ceftriaxone (OB; 15.31, LPG; 6.38) and ciprofloxacin (OB; 12.42, LPG; 5.77). Sulfamethoxazole + trimethoprim was the only originator brand drug with a low MPR, i-e, below 1 (Table 3), which means that retail price of this product is lower than the median bulk procurement price (MSH supplier price) usually offered to the public sector in the developing countries. OBs of other antibiotics with MPR < 2 included co-amoxiclav (1.20), ampicillin (1.50), cefalexin (1.94), metronidazole (1.21) and LPG included amikacin (1.88), amoxicillin (1.87), co-amoxiclav (1.10), azithromycin (1.98), benzyl penicillin (1.79), cefalexin (1.83), cefotaxime (1.47), clarithromycin (1.55), meropenem (1.44) and metronidazole (1.16) (Table 3). The MPRs of these antibiotics showed that they are available at reasonable prices at private pharmacies.

**Table 3:** Individual median price ratios (MPRs) for surveyed antibiotics

Medicine Name	Type	MPR	25%tile	75%tile	Min	Max
Cap. Doxycycline 100 mg *J01AA02*	OB	4.33	4.33	4.33	3.75	4.34
	LPG	2.90	1.70	3.92	1.36	4.94
Cap. Ampicillin 500 mg *J01CA01*	OB	1.50	1.35	1.50	1.25	1.50
	LPG	NA	NA	NA	NA	NA
Cap. Amoxicillin 500 mg *J01CA04*	OB	2.60	2.60	2.61	2.48	2.61
	LPG	1.87	1.51	2.26	1.06	2.59
Inj. Benzyl penicillin 1MIU *J01CE01*	OB	NA	NA	NA	NA	NA
	LPG	1.79	1.66	1.82	1.28	1.92
Tab. Co-amoxiclav 625 mg *J01CR04*	OB	1.20	1.19	1.20	1.19	1.20
	LPG	1.10	1.08	1.11	0.86	1.17
Inj. Piperacillin + tazobactum 4500 mg *J01CR05*	OB	2.45	2.45	2.45	2.34	2.45
	LPG	2.07	2.07	2.07	2.34	2.45
Cap. Cefalexin 500 mg *J01DB01*	OB	1.94	1.92	1.95	1.92	1.99
	LPG	1.83	1.69	1.93	1.53	1.95
Inj. Cefotaxime 1 g *J01DD01*	OB	2.32	2.32	2.32	2.32	2.32
	LPG	1.47	0.88	1.96	0.86	2.01
Inj. Ceftriaxone 1 g inj *J01DD04*	OB	15.31	15.31	15.31	15.31	15.31
	LPG	6.38	6.04	6.38	4.78	8.66
Cap. Cefixime 400 mg *J01DD08*	OB	NA	NA	NA	NA	NA
	LPG	2.17	2.14	2.47	1.32	2.47
Inj. Meropenem 1 g *J01DH02*	OB	NA	NA	NA	NA	NA
	LPG	1.44	1.44	1.80	0.73	2.06
Tab. Sulfamethoxazole + trimethoprim 960 mg *J01EE01*	OB	0.74	0.73	1.48	0.71	1.50
	LPG	NA	NA	NA	NA	NA
Tab.Clarithromycin 500 mg *J01FA09*	OB	2.34	2.34	2.34	1.78	2.38
	LPG	1.55	1.27	1.55	1.16	1.58
Tab. Azithromycin 250 mg *J01FA10*	OB	NA	NA	NA	NA	NA
	LPG	1.98	1.70	2.14	1.55	3.29
Tab. Clindamycin 300 mg *J01FF01*	OB	2.50	2.50	2.54	2.49	3.33
	LPG	NA	NA	NA	NA	NA
Inj. Gentamicin 40 mg/ml *J01GB03*	OB	NA	NA	NA	NA	NA
	LPG	3.04	3.03	3.05	3.02	3.07
Inj. Amikacin 50 mg/ml *J01GB06*	OB	NA	NA	NA	NA	NA
	LPG	1.88	1.87	1.88	1.87	12.44
Tab. Ciprofloxacin 500 mg *J01MA02*	OB	12.42	12.34	12.45	12.15	12.45
	LPG	5.77	5.23	6.81	2.53	6.96
Inj. Vancomycin 500 mg *J01XA01*	OB	4.06	4.06	4.42	3.33	5.37
	LPG	3.73	3.54	4.06	3.42	4.06
Tab. Metronidazole 400 *J01XD01*	OB	1.21	1.21	1.23	1.15	1.41
	LPG	1.16	1.15	1.18	1.15	1.20

*Cap* Capsule, *Tab* Tablet, *Inj* Injection, *MPR* Median Price Ratio

We calculated brand premium (BP)of the drugs whose OBs and LPGs both were available on each one of the surveyed pharmacies. Out of 26 antibiotics, a brand premium of 11 drugs was calculated. The median of brand premium obtained was 38.7% which varied between as low as 3.97% of metronidazole and as high as 140% of ceftriaxone. Notable antibiotics having higher brand premiums include, amoxicillin (MPR; 2.60, B.P; 38.87), cefotaxime (MPR; 2.32, B.P; 57.71), ciprofloxacin (MPR; 12.42, BP; 115.157), ceftriaxone (MPR; 15.31, B.P; 140), clarithromycin (MPR; 2.34, B.P; 51.126), doxycycline (MPR; 4.33, B.P; 49.41) and piperacillin + tazobactum (MPR; 4.06, B.P; 18.63) (Table 4). The drugs with high MPRs show that their prices were significantly above the international reference prices and brand premium was also high of these antibiotics.

**Table 4:** Brand premium (%) of originator brand products compared to lowest price generic equivalents

Medicine name	Brand median price (BMP)	Generic median price (GMP)	Brand premium (%) = [(BMP-GMP)/GMP]*100
Amoxicillin	8.61	6.20	38.87
Co-amoxiclav	21.66	20.00	8.30
Cefalexin	16.87	15.91	6.03
Cefotaxime	276.00	175.00	57.71
Ceftriaxone	672.00	280.00	140.00
Ciprofloxacin	51.10	23.75	115.16
Clarithromycin	65.74	43.50	51.13
Doxycycline	6.35	4.25	49.41
Metronidazole	1.57	1.51	3.97
Piperacillin + tazobactum	955.00	805.00	18.63
Vancomycin	854.50	785.00	8.85

Out of the total, only 6 antibiotics used as standard treatment in clinical conditions i.e. uncomplicated UTI, GIT infection, LRTI, GUTI, CNS and ENT infections were selected for checking their affordability in the private sector. For this purpose, the wage of the lowest paid government worker was used (Annual Budget 2016–2017). The affordability was calculated by comparing median treatment price with a daily wage. Total price of a complete standard treatment surpassing a daily wage was considered unaffordable. The expense on standard treatment with OB is 0.3 day's wage (median) and expense on standard treatment with LPG is 0.25 day's wage (median). Data suggested that treatment with OB and LPG is unaffordable for ciprofloxacin (OB; 6.1, LPG; 2.8) and cefotaxime (OB; 8.3, LPG; 5.2) i.e. more than one day’s wage (Table 5).

**Table 5:** Sensitivity analysis for calculation of affordability using two threshold levels of wages

Indication/ Disease	Antibiotic	Strength	Dosage form	MUP OB	Treatment Duration (in Days)	Total # of Units per day	MTP-OB	Day’s wage for treatment with OB (PKR 304.33*)	Day’s wage for treatment with OB (PKR 594.33*)	MUP LPG	MTP-LPG	Day’s wage for treatment with LPG (PKR 304.33*)	Day’s wage for treatment with LPG (PKR 594.33*)
Uncomplicated UTI	Ciprofloxacin	500 mg	Tablet	51.1	7	2	715.4	2.4	1.2	23.75	332.5	1.1	0.6
Amoebiasis/Giardiasis GIT	Metronidazole	400 mg	Tablet	1.57	10	3	47.1	0.2	0.1	1.51	45.3	0.1	0.1
LRTI	Amoxicillin 500 mg	500 mg	Capsule	8.61	10	2	172.2	0.6	0.3	6.2	124	0.4	0.2
GUTI	Doxycycline	100 mg	Capsule	6.35	7	2	88.9	0.3	0.1	4.25	59.5	0.2	0.1
CNS	Cefotaxime	1000 mg	Injection	276	7	2	3864	12.7	6.5	175	2450	8.1	4.1
ENT	Amoxicillin	500 mg	Capsule	8.61	5	3	129.15	0.4	0.2	6.2	93	0.3	0.2

*ENT* Ear Nose Throat Infection, *UTI* Urinary Tract Infection, *GIT* Gastrointestinal Tract Infection, *GUTI *Genito-urinary Tract Infection, *CNS* Central Nervous System Infection, *LRTI *Respiratory Tract Infection, *Tab * Tablet, *Inj* Injection, *Cap* Capsule, *MUP* Median unit price; *MTP* Median treatment price

* Minimum and maximum daily wage of lowest paid government worker in Pakistan (2017)

### Discussion

Unaffordable prices and low availability of medicines are key barriers in providing medical care to the patients [26]. The prices and availability can only be controlled by developing proper pricing policies and by ensuring their implementation. In Pakistan, under DRAP Act 2012, drug pricing committee (DPC) controls and regulates medicine prices. Although, the DRAP has devised two drug pricing policies, one in 2015 and the other in 2018, in order to define the strategies to rationally calculate the MRPs i.e. suitable for both patients and manufacturers. But unfortunately, these strategies could not produce remarkable results, especially for patients. Most of the essential medicines are still unaffordable in Pakistan [27] and it could lead to non-adherence. In 2005, access to essential medicines was first evaluated in Pakistan, by Kiani et al., by using standard WHO/HAI methodology [28]. Afterwards, the WHO/HAI methodology was updated in 2008, and Amna et al. measured the access to essential medicines in Lahore division, Pakistan, while using the updated methodology [16]. The aforementioned studies reported poor availability and affordability of essential medicines from 2005 to 2017. To the best of our knowledge, not a single study has been conducted focusing on one specific class of drugs i.e. only a few drugs were included from each category. So, this is the first study from Pakistan that was carried out in private sector to evaluate the prices, availability and affordability of selected key access antibiotics of essential medicines list 2017 adopted by WHO, by using a variant of WHO/HAI methodology [29]. Only the private sector was chosen because in the majority of public sector facilities the medicines are provided free of cost to the patients, and due to poor availability in public sector, patients often go to the private retail pharmacies to get medicines further adding to their out of pocket expenses [30]. Out of the total, only 5 key access antibiotics were available in all pharmacies. WHO has classified antibiotics into access, watch and reserve group in order to enhance treatment outcome and reduce the burden of AMR. Key access antibiotics should be widely available, affordable and quality-assured because these antibiotics are first and second choice antibiotics for most common general infections [31]. Poor availability of key access antibiotics may be due to budgetary issues, demand for watch group antibiotics or delays in the distribution of key antibiotics all leading to stock-outs [32]. In the current study, the availability of LPG of amoxicillin was only 37.5% as compared to 93.8% of OB. On the other hand, availability of more potent broad-spectrum antibiotics, ciprofloxacin (OB; 93.8%, LPG; 100%) and co-amoxiclav (OB, 100%, LPG; 68.8%), was much higher. Unavailability of key access antibiotics can lead to inappropriate prescribing of antibiotics or even resulted in wrong choice, wrong dose, poor quality drug or delays in the treatment [33]. This could also result in a rapid increase of AMR to extended-spectrum beta-lactams and quinolones [34].

The literature reports indicate that MPR value less than or equal to 5 is considered acceptable in private sector pharmacies [35]. The current data depicted very high MPR of ceftriaxone (OB; 15.31, LPG; 6.04) and ciprofloxacin (OB; 12.42, LPG; 5.77) in the private sector as compared to other antibiotics. However, MPR of doxycycline, amoxicillin and vancomycin was also greater than 2.5. Whereas, only Sulphamethoxazole + trimethoprim in OB category has MPR below 1, showing lower retail price than international reference price. Moreover, the median brand premium was 38.87% and in some cases, it is as high as 140% for ceftriaxone and 115.157% for ciprofloxacin. This indicated higher prices of OBs whereas the preferred limit according to drug pricing policy of Pakistan is 30% for the drugs having more than three generic equivalents in the market [36]. Drug pricing committee must take steps to limit, review and regulate the prices of both OB and LPG of antibiotics so that the treatment expenses for a patient become affordable. The monopoly of OB manufactures can be prevented by promoting generic prescription as well as generic substitution. Generic prescribing offers the opportunity for major savings to the consumers and government in healthcare expenditure [37, 38].

Data also indicated LPGs are more affordable than OBs and their affordability can further be increased by maintaining proper mark ups [39]. Immediate actions are required to improve medicine affordability, either by promoting the use of good quality, low-priced generics, and by launching health insurance systems [40]. Poor availability and unaffordability of key access antibiotics may also increase antimicrobial resistance in the community by promoting non-adherence to therapy [41–43]. The potential emergence of resistance due to non-adherence to recommended therapeutic regime, for antibiotics sharing the status of key ACCESS antibiotics with WATCH group (discussed earlier) is much more. Table 1 show that relatively better availability status for LPGs for WATCH/ACCESS group antibiotics whereas it can be observed in Table 5 that the affordability of this subgroup is low, especially for ciprofloxacin and cefotaxime, the two antibiotics from this subgroup.

High availability and low affordability is more indicative of the fact that patients might be started on the treatments but it might not be possible for them to complete the antibiotics course using standard treatment regime. The interventions promoting generic medicines have been proven useful and the educational programmes are needed for healthcare professionals and consumers to promote generic medicines in Pakistan [44, 45]. Refining the governance and supervision competence, and evaluating local supply possibilities, may improve availability. Prices could be condensed by improving purchasing efficiency, excluding taxes and regulating mark-ups [46].

There are a few limitations of the study as well. The availability of medicines is only depicting the data available on the day data was collected and not depicting the average availability of medicines. All pharmacies are not expected to carry all EML medications, particularly ones that see infrequent/niche use such as chloramphenicol and spectinomycin. Additionally, the comparison is made by only relying on the median price ratios that depend on supplier prices which are used to find out the median international reference price. When supplier prices are not available and a buyer price is used as proxy then results of median price ratio may vary depending on changes in international reference prices [47].

The affordability was estimated considering government lowest daily wage worker’s salary that might represent an over-estimation of affordability, since many private workers earn less than government daily wage worker. Since it’s a variant of WHO/HAI standard methodology and is conducted on a city level (not countrywide) there is room for further improvement in getting stronger evidence. A small sampling of pharmacies at a single site in Pakistan may not be representative of the country owing to its diverse geographical, cultural and socio-economic settings. Provincial trends for example, in Baluchistan or Sindh provinces may drastically vary from those in the Punjab. Similarly, the metropolitan cities, urban and rural divide may also be reflected in the results from a larger and broader sampling. Before making any national policy recommendations, itis advisable to collect representative sampling in these regions. Therefore, we can further evaluate in future if the EML medication unavailable at private pharmacies (cloxacillin, cefazolin, nitrofurantoin, etc.) are available in the public sector. Perhaps these medications are not available at private pharmacies because they are available free of cost by public pharmacies and therefore are not cost-effective to maintain privately [21].

## Conclusion

The prices and availability of OB were relatively higher as compared to LPG. The higher availability of comparatively costlier OB shows their frequent prescription by physicians. Promotion of good quality generic medicines can solve this issue. Thus, despite the already present pricing policies, there is still a need for improvement in these policies as many of the drug treatment prices surpass the minimum daily wage. There is a need for proper policies and assurance of their implementation so that the availability of both OB and LPG of key access antibiotics should be increased. More literature evidence should be made available on the latest pricing, availability and affordability of key access antibiotics of WHO EML. By making and launching better policies these problems can be overcome. Prices could be reduced by improving purchasing efficiency, excluding taxes and regulating mark-ups and thus increasing the affordable range for the patients to complete their antibiotic therapy and subsequently reduce antimicrobial resistance.

## Data Availability

All the data supporting the conclusion of this article are included within the article.

## References

[1] Vasheghani Farahani A, Salamzadeh J, Rasekh HR, Najafi S, Mosadegh V. The Availability and Affordability of Cardiovascular Medicines for secondary prevention in Tehran Province (Winter Special Issue 2018). Iranian Journal of Pharmaceutical Research. 2018. PMC5958325 29796030

[2] Babar ZUD, Ibrahim MIM, Singh H, Bukahri NI, Creese A (2007). Evaluating drug prices, availability, affordability, and price components: implications for access to drugs in Malaysia. PLoS Med.

[3] Abiye Z, Tesfaye A, Hawaze S. Barriers to access: availability and affordability of essential drugs in a retail outlet of a public health center in south western Ethiopia. 2013.

[4] Alsairi R (2017). Access to Medicine in Developing Countries. Am J Med Medical Sci.

[5] Davies J, Davies D (2010). Origins and evolution of antibiotic resistance. Microbiol Mol Biol Rev.

[6] Marquet K, Liesenborgs A, Bergs J, Vleugels A, Claes N (2015). Incidence and outcome of inappropriate in-hospital empiric antibiotics for severe infection: a systematic review and meta-analysis. Crit Care.

[7] Gould I (2009). Antibiotic resistance: the perfect storm. Int J Antimicrob Agents.

[8] Organization WH. World Health Assembly addresses antimicrobial resistance, immunization gaps and malnutrition. World Health Organization Geneva; 2015.

[9] Cars O, Hedin A, Heddini A (2011). The global need for effective antibiotics—moving towards concerted action. Drug Resist Updates.

[10] Laing R, Waning B, Gray A, Ford N, Hoen E (2003). 25 years of the WHO essential medicines lists: progress and challenges. Lancet.

[11] Zaidi S, Nishtar N. Access to essential medicines: in Pakistan identifying policy research and concerns. 2011. http://www.haiweb.org/medicineprices/surveys/200407PK/survey_report.pdf.

[12] Kiani A, Qadeer A, Mirza Z, Khanum A, Tisocki K, Mustafa T. Prices, availability and affordability of medicines in Pakistan. Report of the network for consumer protection, Islamabad Accessed. 2010;10.

[13] Hsia Y, Lee BR, Versporten A, Yang Y, Bielicki J, Jackson C (2019). Use of the WHO Access, Watch, and Reserve classification to define patterns of hospital antibiotic use (AWaRe): an analysis of paediatric survey data from 56 countries. Lancet Global Health.

[14] Organization WH. The selection and use of essential medicines: report of the WHO Expert Committee, 2017 (including the 20th WHO Model List of Essential Medicines and the 6th Model List of Essential Medicines for Children): World Health Organization; 2017.

[15] Pakistan DRAo, Ministry of National Health Services, Coordination Ra, Pakistan Go. National essential medicines list 2018. national essential medicines list 2018. 2018.

[16] Saeed A, Saeed H, Saleem Z, Fang Y, Babar ZUD (2019). Evaluation of prices, availability and affordability of essential medicines in Lahore Division, Pakistan: A cross-sectional survey using WHO/HAI methodology. PLoS ONE.

[17] Saleem Z, Hassali MA, Hashmi FK, Godman B, Saleem F. Antimicrobial dispensing practices and determinants of antimicrobial resistance: a qualitative study among community pharmacists in Pakistan. Family Medicine and Community Health. 2019. 10.1136/fmch-2019-000138 PMC6910752 32148717

[18] THE DRUGS (LICENSING RAAR, 1976. THE DRUGS (LICENSING, REGISTERING AND ADVERTISING) RULES, 1976.

[19] Organization WH. Measuring medicine prices, availability, affordability and price components. 2008.

[20] Organisation WH. 20th Essential Medicines List (2017) 2017 [cited 2018 May 20]. http://www.who.int/medicines/news/2017/20th_essential_med-list/en/.

[21] Dawani K, Sayeed A. Pakistan’s pharmaceutical sector: issues of pricing, procurement and the quality of medicines. 2019.

[22] International Medicines Price Workbook (Version 6.1). International Medicines Price Workbook (Version 6.1),

[23] International Medical Products Price Drug 2015 Boston: Management Sciences for Health; 2015 [cited 2019 May 11]. http://mshpriceguide.org/en/home/.

[24] Gelders S, Ewen M, Noguchi N, Laing R. Price, availability and affordability. An international comparison of chronic disease medicines. Price, availability and affordability An international comparison of chronic disease medicines: OMS/HAI; 2006.

[25] https://paycheck.pk/salary/minimum-wages/public-sector. https://paycheck.pk/salary/minimum-wages/public-sector.

[26] Committee JF, BRBnfPP. (2017). Committee JF, Britain RPSoG.

[27] Saeed A, Saeed H, Saleem Z, Yang C, Jiang M, Zhao M (2020). Impact of National Drug Pricing Policy 2018 on access to medicines in Lahore division, Pakistan: a pre-post survey study using WHO/HAI methodology. BMJ open.

[28] Kiani A, Qadeer A, Mirza Z, Khanum A, Tisocki K, Mustafa T. Prices, availability and affordability of Medicines in Pakistan. Report of The Network for Consumer Protection. 2007.

[29] http://www.who.int/medicines/news/2017/20th_essential_med-list/en/. OWtEMLcMAf. Organisation WH. 20th Essential Medicines List (2017) 2017 [cited 2018 May 20]. Available from: http://www.who.int/medicines/news/2017/20th_essential_med-list/en/.

[30] Lessing C, Mace C, Bissell K (2013). The availability, pricing and affordability of three essential asthma medicines in 52 low-and middle-income countries. Pharmacoeconomics.

[31] https://www.who.int/news/item/06-06-2017-who-updates-essential-medicines-list-with-new-advice-on-use-of-antibiotics-and-adds-medicines-for-hepatitis-c-hiv-tuberculosis-and-cancer. https://www.who.int/news/item/06-06-2017-who-updates-essential-medicines-list-with-new-advice-on-use-of-antibiotics-and-adds-medicines-for-hepatitis-c-hiv-tuberculosis-and-cancer.

[32] Patel A (2007). Prices & availability of common medicines at six sites in India using a standard methodology. Indian J Med Res.

[33] https://accesstomedicinefoundation.org/publications/shortages-stockouts-and-scarcity-the-issues-facing-the-security-of-antibiotic-supply-and-the-role-for-pharmaceutical-companies. https://accesstomedicinefoundation.org/publications/shortages-stockouts-and-scarcity-the-issues-facing-the-security-of-antibiotic-supply-and-the-role-for-pharmaceutical-companies. acess to medicine foundation. 2018.

[34] Bartoloni A, Pallecchi L, Riccobono E, Mantella A, Magnelli D, Di Maggio T (2013). Relentless increase of resistance to fluoroquinolones and expanded-spectrum cephalosporins in Escherichia coli: 20 years of surveillance in resource-limited settings from Latin America. Clin Microbiol Infect.

[35] Gelders S, Ewen M, Noguchi N, Laing R (2006). Price, availability and affordability: an international comparison of chronic disease medicines.

[36] http://www.dra.gov.pk/userfiles1/file/Drug%20Pricing%20Policy%202015.pdf. http://www.dra.gov.pk/userfiles1/file/Drug%20Pricing%20Policy%202015.pdf.

[37] Kanavos P (2007). Do generics offer significant savings to the UK National Health Service?. Curr Med Res Opin.

[38] Kalisch LM, Roughead EE, Gilbert AL (2007). Pharmaceutical brand substitution in Australia–are there multiple switches per prescription?. Aust N Z J Public Health.

[39] Mhlanga BS, Suleman F (2014). Price, availability and affordability of medicines. African J Primary Health Care Family Med.

[40] Niëns L, Cameron A, Van de Poel E, Ewen M, Brouwer W, Laing R (2013). Quantifying the impoverishing effects of purchasing medicines: A cross-country comparison of the affordability of medicines in the developing world. Alexandra Cameron.

[41] Carey B, Cryan B (2003). Antibiotic misuse in the community–a contributor to resistance?. Irish Med J.

[42] Goossens H, Ferech M, Vander Stichele R, Elseviers M, Group EP (2005). Outpatient antibiotic use in Europe and association with resistance: a cross-national database study. Lancet.

[43] Kardas P (2002). Patient compliance with antibiotic treatment for respiratory tract infections. J Antimicrob Chemother.

[44] Babar ZUD, Kan S, Scahill S (2014). Interventions promoting the acceptance and uptake of generic medicines: a narrative review of the literature. Health Policy.

[45] Jamshed SQ, Babar Z, Ibrahim M, Hassali M (2009). Generic medicines as a way to improve access and affordability: a proposed framework for Pakistan. J Clinical Diagnostic Res.

[46] Mendis S, Fukino K, Cameron A, Laing R, Filipe A, Khatib O (2007). The availability and affordability of selected essential medicines for chronic diseases in six low-and middle-income countries. Bull World Health Organ.

[47] Cameron A, Ewen M, Ross-Degnan D, Ball D, Laing R (2009). Medicine prices, availability, and affordability in 36 developing and middle-income countries: a secondary analysis. Lancet.

